# Associations and interactions between prokaryotes and other gut biota in non-human primates

**DOI:** 10.1007/s10329-025-01224-1

**Published:** 2025-11-18

**Authors:** Marina Bambi, Heidi C. Hauffe, Claudia Barelli

**Affiliations:** 1 Department of Biology, University of Florence, Via Madonna del Piano 6, Sesto Fiorentino, 50019 Florence, Italy; 2 Conservation Genomics Research Unit, Research and Innovation Centre, Fondazione Edmund Mach, Via E. Mach 1, 38098 San Michele all’Adige, Italy; 3 National Biodiversity Future Center (NBFC), Palermo, Italy

**Keywords:** Bacteria, Fungi, Helminths, Host health, Literature review, Viruses

## Abstract

The influence of gut microbiota on vertebrate health, development, growth, and physiology is increasingly evident. Considerable research has concentrated on Primates, not only because many non-human species have long acted as laboratory models to better understand human health, but also to improve our knowledge of the ecology of this mammalian Order with the highest proportion of endangered wild taxa. While bacteria have been the primary focus, other gut components (archaea, protozoa, fungi, viruses, and helminths) also play critical roles in host survival. Only recently have there been microbiota studies examining interactions among gut components and their influence in both captive and wild non-human primates. Here we systematically reviewed all research articles on non-human primates where gut bacteria were studied together with at least one other gut taxon. Studies on humans were included in each comparison where these suggested (i) potential health outcomes of patterns observed in non-human primates, or (ii) how approaches used in human microbiota studies could more effectively advance non-human primate research and conservation. Specifically, we focused on associations (such as covariations in diversity indices or abundance between taxa), direct interactions (one taxon affecting another with experimental evidence), and external influences (e.g., disease, diet, environment) impacting multiple taxa. We found that for non-human primates, direct interactions among bacteria and other gut taxa have rarely been confirmed except under experimental captive conditions, especially for wild species. However, the associated changes in richness, abundance, and/or metabolic pathways indicate interdependence among gut taxa, confirming their links with host nutrition and immunity and, consequently, their potential impact on host health and species conservation.

**Supplementary Information:**

The online version contains supplementary material available at 10.1007/s10329-025-01224-1.

## Introduction

The intestinal tract of all animal species is colonized by a wide variety of micro- (bacteria, archaea, protozoans, fungi and viruses) and macro- (helminths) organisms, all of which play vital roles in gut health. For instance, bacteria have been shown to promote host well-being by fermenting food compounds and converting them into unique metabolites that support other organs (Costello et al. 2009; Puupponen-Pimiä et al. 2002). Because they have co-evolved within their hosts for millions of years, these gut microorganisms are highly likely to affect not only the host, but also each other (Cheng et al. 2019; Krautkramer et al. 2020; Vemuri et al. 2020). Therefore, the animal gut offers a suitable model to explore whether the various components of the host gut interact, and how such ‘crosstalk’ affects host health (Kau et al. 2011; Shi et al. 2017).

Gut bacteria are well-known to contribute to host development, metabolism, immunity, growth and behaviour (Hooper et al. 2012; Rooks and Garrett 2016; Schwarzer et al. 2018). In fact, they are the most extensively studied component of the gut microbiota, largely because methods and technologies for their investigation have long been available and research has historically concentrated on them. However, more recent research indicates that other gut taxa may play important roles in host health as well. For example, fungi (mycobiota) are involved in host metabolic function and immunity (Chin et al. 2020; Gutierrez and Arrieta 2021; Gutierrez et al. 2025), while the absence of helminths and protozoans in the gut has been associated with auto- and hyper-immune diseases in humans (Bilbo et al. 2011; Loke and Lim 2015; Carrera Silva et al. 2025). Moreover, although commonly considered agents of morbidity and mortality, viruses and bacteriophages (viruses infecting bacteria) also appear to be key regulators of bacterial communities and processes (Guerin and Hill 2020; Mills et al. 2013; Mirzaei and Maurice 2017; Nayfach et al. 2021).

The above observations suggest that bacteria are not the only essential players in gut homeostasis; in addition, the interactions between gut components may also be relevant to understanding the implications any change may cause on overall host health (Filyk and Osborne 2016; Liu et al. 2022; Akiyama et al. 2024). For example, antibiotic treatments in humans change not only the richness (number of taxa) and composition (relative abundance of taxa) of targeted bacteria, but also cause a decrease in the abundance of beneficial fungal taxa, allowing potentially pathogenic mycobiota to flourish (i.e., *Candida* spp.; Seelbinder et al. 2020; Ventin-Holmberg et al. 2022). Furthermore, the transmission routes by which these micro- and macro-organisms are acquired by the host (e.g., fecal–oral, trophic, skin penetration, or close-contact transmission) may shape their relationships with the host and with each other, as routes differing in persistence, exposure intensity, and host colonization could lead to distinct ecological outcomes.

However, results from human studies may not accurately reflect microbiota interactions in wild primates. Non-human primate species, for instance, have diverse diets that change seasonally, leading to more frequent microbiota shifts (Li et al. 2023; Sawada et al. 2022). Additionally, their frequent exposure to soil through behaviours such as deliberate ingestion (geophagy) and grooming after contact with ground surfaces introduces a variety of microorganisms including intestinal parasites, further shaping microbiota composition and interactions (Barelli et al. 2021; Grieneisen et al. 2019).

Given its potential importance to individual health and species survival, gut microbiota research is considered an increasingly relevant topic in animal conservation (Barelli et al. 2021; Hauffe and Barelli 2019; Pascoe et al. 2017; Trevelline et al. 2019). Therefore, understanding interactions between the components of gut micro-biodiversity may serve to understand their potential as non-invasive bioindicators of health status especially in threatened or endangered populations.

In fact, Primates are the mammalian Order with the highest percentage of species assessed as critically endangered (IUCN 2025), and studies on the diversity and dynamics of their intestinal microorganisms are steadily increasing both in the wild and in captivity (Clayton et al. 2018; Nishida and Ochman 2021; Dallas and Warne 2023). However, a systematic literature review of whether and how individual gut components interact has not been carried out thus far. Our aims here were to complete such a review, assess if and how the variation of one gut component affects or potentially affects another, and evaluate the potential implications of these interactions for host health. Here we collected scientific articles up to January 2025 that focused on gut bacteria and at least one other gut taxon in non-human primates. We selected articles in which associations (i.e., statistical co-variations in diversity indices or abundance between different gut taxa) and/or direct interactions (i.e., changes in one taxon has been experimentally and statistically shown to affect another) were tested, as well as studies in which the influence of external factors such as disease, diet, or environment on more than one microbiota component were studied.

Specifically, we used evidence from the selected scientific articles to examine and discuss: (a) the pairwise associations or interactions of bacteria with all other gut taxa; (b) the effects of such associations or interactions on the host immune system and overall health; and (c) the intrinsic and extrinsic factors impacting these associations or interactions and their role in the observed outcomes.

## Methods

In this analysis, only peer-reviewed scientific articles on non-human primates, both captive and wild, that focused *simultaneously* on gut bacteria and another gut taxon were included in the systematic search. Relevant studies on humans that provided simultaneous investigations were also included to complement the evidence available for non-human primates, offering comparative insights and highlighting potential outcomes and future directions in non-human primate microbiota research.

Google Scholar was initially used to search for journal articles published in English and sorted by relevance up to January 2025, using those satisfying at least one of the following strings:

"*Gut bacteria OR microbiome OR microbiota AND [TAXON] AND primates OR monkeys*" where [TAXON] equalled: fungi OR mycobiota OR mycobiome; eukaryotes; helminths OR soil-transmitted helminths; gut parasites OR gastrointestinal parasites; protozoans; or viruses.

For each of these six searches, the first 100 articles were downloaded and checked for redundancy, resulting in a total of 453 unique articles. Articles on gut microbiota that only described taxonomic diversity without reporting associations/interactions or that studied multiple taxa without highlighting relationships were excluded (N = 369), leaving 84 articles for consideration. The same strings were then used on the Web of Science to confirm that no relevant articles were missing. This search retrieved an additional 34 unique articles of which 24 were excluded since did not meet inclusion criteria (i.e. simultaneous analyses of bacteria and another taxon providing evidence of associations or interactions), leaving 10 articles for consideration.

For each pairwise comparison of gut components, we summarized research findings to date, highlighting how these associations/interactions are known to influence the gut ecosystem and, consequently, the health and adaptability of the host. Specifically, we analyzed 57 studies conducted in non-human primates (25 for viruses, 12 for fungi, 11 for helminths, and nine for protozoa; Table S1) and 40 studies in humans (10 articles for viruses, 14 for fungi, seven for helminths, and nine for protozoa; Table S2) for a total of 94 articles. A full list of excluded studies is also provided (Table S3). In this way, we provide a comprehensive overview of the dynamic interplays within the non-human primate gut microbiota and its implications for host health and conservation.

## Results/Discussion

### Bacteria and protozoa

Gut protozoa include both pathogenic and non-pathogenic species; however, those that have been most extensively studied are primarily known for their pathogenic potential and are often transmitted between hosts by a fecal–oral route (Mak 2004), including all protozoa examined in wild non-human primates in this review. However, transmission risk and community composition are also likely influenced by ecological factors such as habitat use or dietary diversity. For instance, in chimpanzees (*Pan troglodytes*), the composition and richness of both gut protozoa and bacteria varied across sites, with higher diversity of both gut components in forested habitats richer in plant resources (Bueno de Mesquita et al. 2021). Such findings suggest that ecological filtering (i.e., through differences in diet and habitat) may affect both protozoan acquisition (via co-exposure to shared environmental sources) and their associations with other gut microbiota components.

Thus far, studies focusing on protozoa with pathogenic potential generally report negative effects on gut and host health; for example, causing enteric infections in humans, especially in countries where sanitation and water treatment are inadequate (Speich et al. 2016). However, protozoa may also play alternative roles and occur in association with healthy hosts (Chabé et al. 2017). In non-human primates, their presence has been linked to changes in bacterial richness or composition, with outcomes in protozoa varying across species. For example, in *Eimera*-positive mouse lemurs (*Microcebus rufus*) intestinal bacterial richness was higher, indicating greater gut microbiota diversity compared to non-infected individuals, although the implications for host gut health remain unclear (Aivelo and Norberg 2018).

On the other hand, Coquerel sifaka (*Propithecus coquereli*) infected with *Cryptosporidium* (McKenney et al. 2017), rhesus macaques (*Macaca mulatta*) infected with *Plasmodium cynomolgi* (Farinella et al. 2023) and black and gold howler monkeys (*Alouatta caraya*) infected with *Giardia* spp. (Kuthyar et al. 2021) all showed a substantial decrease in bacterial richness, which in these cases results in adverse health effects, such as the reduction of beneficial bacterial families and a shift to dysbiosis, which increases the susceptibility of the gut to colonization by pathogenic microorganisms.

The protozoan genera *Entamoeba* and *Blastocystis* are the most frequently studied in humans and non-human primates due to their high prevalence, medical importance, and zoonotic potential (Vaisusuk and Saijuntha 2021). Their capacity to infect a broad range of hosts makes these two protozoa good models for exploring the complexity of the dynamics that influence their pathogenicity and relationships with other intestinal components. For example, the presence of *Entamoeba* spp. is associated with an increased abundance of bacterial families with beneficial roles in nutrition and metabolism (e.g., *Erysipelotrichaceae* and *Anaeroplasmataceae*), as observed in wild western lowland gorillas (*Gorilla gorilla gorilla*) (Vlčková et al. 2018), and those with a protective role against infection (e.g., *Lachnospiraceae*), as observed in several wild non-human primates (five platyrrhines, six cercopithecoids, three lemurs and two monkeys; Mann et al. 2020). In contrast, *Blastocystis* spp. affects the gut microbiota of non-human primates in less predictable ways. For example, in wild western chimpanzees (*P. troglodytes verus*), *Blastocystis* spp. is linked to a decrease in beneficial bacteria (*Faecalibacterium*) and *Bacteroides*-associated enterotypes, instead promoting potentially pathogenic genera (e.g., *Oscillibacter, Prevotella_1, Peptococcus*). However, it is also associated with increased abundance of the anaerobic archaea *Methanobrevibacter*, which is believed to support gut health (Renelies-Hamilton et al. 2019). On the other hand, captive-reared and diseased douc langur (*Pygathrix nemaeus*), characterized by chronic vomiting and diarrhea, had significantly lower relative abundance of *Blastocystis*, combined with an increase in some potential pathogenic bacteria (e.g., *Campylobacter*) and a reduction in beneficial ones (e.g., *Akkermansia*) compared to healthy douc (Amato et al. 2016), suggesting a protective role for *Blastocystis*. Such mixed results are also noted in a number of papers on human microbiota; for example, Cameroonians positive for *Entamoeba* spp. had greater intestinal bacterial richness (Even et al. 2021; Morton et al. 2015), but patients colonized specifically by *E. histolytica* suffered a reduction in beneficial bacteria (Iyer et al. 2023); *Blastocystis*-positive patients showed greater bacterial richness (Andersen et al. 2015; Audebert et al. 2016; Even et al. 2021; Forsell et al. 2017; Tito et al. 2019), or positive changes in bacterial composition, such as greater abundance of as *Lachnospiraceae* and *Eggerthellaceae* (Beghini et al. 2017; Geng et al. 2024). Most of these findings come from observational studies based on targeted 16S rRNA profiling, which generally only identifies taxa to the genus level, limiting the ability to infer functional or clinical significance. An exception is the shotgun metagenomics study by Forsell et al. (2017), which also assessed clinical symptoms and found no association between *Blastocystis* presence and gastrointestinal illness in humans, suggesting that this protozoan may be associated with a richer and more stable bacterial community. However, even in this case, causality cannot be established.

Overall, these results appear to confirm that protozoa and bacteria, as well as archaea, may affect each other’s diversity, but the results published thus far do not confirm direct interaction. However, what is clear is that protozoans should not always be considered pathogenic, as they may also have protective roles. To date, there are no studies focusing on whether intestinal prokaryotes can prevent colonization by pathogenic protozoans, or whether protozoans associated with increases in intestinal bacterial and archaeal diversity achieve these changes by interacting indirectly with the host immune system, or directly with prokaryote communities. Further investigations are needed to examine the significance and health implications of these potential interactions.

### Bacteria and helminths

Parasitic helminths (trematodes, cestodes, nematodes and acanthocephalans) are a polyphyletic group of macro-parasitic intestinal worms that infect the gastrointestinal tract through a variety of different transmission routes (Walusimbi et al. 2023). Some species require environmental development and spread via fecal–oral ingestion of embryonated eggs (e.g., *Trichuris, Lemuricola, Callistoura, Trypanoxyuris, Enterobius*), while others rely on intermediate hosts for transmission (e.g., *Hymenolepis diminuta*, *Controrchis biliophilus*). By contrast, certain parasites are capable of autoinfection, completing their life cycle entirely within a single host. These include *Strongyloides stercoralis*, which reinfects via mucosal or perianal skin penetration, and *Hymenolepis nana*, which can bypass the need for an intermediate host. The wide diversity of helminth species and natural histories has led to enormous variation in gut infections and co-infections, which like bacteria and protozoa, may be specific to certain regions. For instance, in western lowland gorillas, helminth diversity varies geographically, with higher strongylid richness reported in populations inhabiting lowland tropical forests, likely reflecting ecological filtering linked to habitat type, altitude, and plant diversity (Mason et al. 2022). However, such patterns are not universal, as demonstrated in a study of 12 lemur species where no association with habitat was noted (Donohue et al. 2023).

Although our search did not identify any studies examining the interactions between archaea and helminths in the gut of non-human primates, numerous investigations have explored the relationships between helminths and gut bacteria. The presence of certain helminth taxa has been linked to changes in bacterial richness, such associations may reflect shifts in the abundance of specific bacterial gut flora, and alterations in bacterial composition. Indeed, an increase in bacterial richness is common in helminth-positive humans and non-human primates and is often interpreted as a sign of gut stability, that is, a microbial community with greater resilience and functional redundancy. However, this may not necessarily be interpreted as direct evidence of a healthier gut, especially considering that most available data come from observational studies, which limit conclusions regarding causality and functional significance. For example, lemur species infected with *Lemuricola* spp. (de Winter et al. 2020), and Udzungwa red colobus monkeys (*Piliocolobus gordonorum*), and yellow baboons (*Papio cynocephalus*) positive for *Trichuris* spp. (Barelli et al. 2021) exhibited increased gut bacterial richness, suggesting potential health benefits. Similar patterns were observed in humans, where helminth-positive individuals from various geographical regions including Malaysia (Lee et al. 2014), Liberia and Indonesia (Rosa et al. 2018), Cameroon (Rubel et al. 2020), and Italy (Jenkins et al. 2018) had greater bacterial richness compared to those without helminths or treated with anthelmintics. In some cases, experimental infections with *Necator americanus* (hookworm) in patients with inflammatory disorders like celiac disease were associated with a significant increase in bacterial species richness and improved gluten tolerance (Giacomin et al. 2015). In others, increased diversity co-occurred with elevated inflammatory markers (Rubel et al. 2020). Conversely, reduced bacterial richness and abundance were observed in geladas (*Theropithecus gelada*) infected with the strongylid nematode *Oesophagostomum* spp., which coincided with elevated levels of neopterin, a marker of inflammation and immune activation (Schneider-Crease et al. 2022), confirming that certain helminths and/or gut bacteria may modulate the inflammatory response.

With regards to abundance, in howler monkeys, helminth infections (such as *Controrchis biliophilus*, the unidentified *Trematode*, *Strongyloides* sp., and *Enterobius* sp.) were associated with changes in the relative abundances of the Orders *Clostridiales* and *Bacteroidales* across habitats varying in fragmentation, disturbance, and diet composition, with higher helminth diversity and more stable microbiota in intact and undisturbed forests (MacFarland 2021). In olive baboons (*P. anubis*), strongylid nematodes were linked to decreased levels of *Prevotella*, a pattern observed in savanna populations under marked seasonal and dietary shifts (Cizauskas et al. 2022). In red-fronted lemurs (*Eulemur rufifrons*), unspecified helminths were associated with an increased abundance of *Verrucomicrobiota* and decreased *Lachnospiraceae*y, coinciding with strong seasonal variation in rainfall, food availability, and stress hormone levels (Murillo et al. 2022). Similarly, in mouse lemurs, the cestodes *Hymenolepis diminuta* and *H. nana* were linked to opposite effects on bacterial orders, affecting both beneficial (*Lactobacillales*, *Bacteroidales*), and disease-causing (*Clostridiales, Enterobacteriales, Burkholderiales, Fusobacteriales*) taxa (Aivelo and Norberg 2018), in different microhabitats and across seasons. Taken together, these findings suggest that the associations between helminths and bacteria observed in wild non-human primates are also the result of distinct ecological factors (such as seasonal variation, habitat type, and diet composition), although direct interactions between the two taxonomic groups cannot be ruled out.

However, in all the above studies, even though many of the associations include taxonomic orders of bacteria, there were no clear indications of the impacts of these changes on host health, except one, where rhesus macaques with chronic idiopathic diarrhea exhibited reduced levels of immune-stimulating bacteria following artificial infection with *Trichuris* spp., highlighting potential impacts of helminths on inflammation-promoting bacterial genera (Broadhurst et al. 2012).

Variation in gut bacterial composition in association with helminth presence has also been documented in non-human primates. For instance, changes in bacterial composition in association with *Trypanoxyuris* spp. and *Callistoura* spp. were observed in wild black howler monkeys (Martínez-Mota et al. 2021) and four lemur species (de Winter et al. 2020), respectively. Again, these studies give no clear indications of how such changes could affect host health; in fact, many associations include both beneficial and disease-causing bacteria. In contrast, human studies frequently report the health implications of helminth-driven changes in bacterial composition. For instance, the presence of helminth infections (such as *Ascaris lumbricoides, Necator americanus, Heligmosomoides polygyrus, Trichuris trichiura and T. muris*) in humans is purported to be associated with increased levels of anti-inflammatory bacteria (*Akkermansia municipalia*: Jenkins et al. 2017; Bacteroidales: Lee et al. 2014; Rubel et al. 2020; *Olsenella*: Rosa et al. 2018), as well as potentially pathogenic bacteria such as *Bacteroides eggerthii* (Jenkins et al. 2017, 2018). Conversely, beneficial bacteria (e.g., *Bacteroides, Bifidobacterium, Lactobacillus, Clostridium*) are often seen to decrease during helminth infections (such as *Fasciola hepatica, Ascaris lumbricoides, Trichuris trichiura e Taenia spp.*) in humans (Silva-Caso et al. 2024).

As noted above, non-human primate studies are largely observational and lack experimental evidence to clarify causal relationships or health impacts. By contrast, human studies often provide insights into the implications of helminth-bacterial associations for host health. In fact, multiple studies on the human gut microbiota have analyzed changes in functional metabolic pathways associated with helminth infections, using advanced shotgun sequencing or by investing in analyses to quantify specific metabolites (using, for example, mass spectrophotometry). These studies have revealed significant variations in microbial metabolism that are strongly associated with the host immune system. For instance, volunteers from northern Italy infected with *Strongyloides stercolaris* showed a lower abundance of short-chain fatty acids (SCFAs) with anti-inflammatory properties (e.g., butyrate) and increased amino acid production (Jenkins et al. 2018). In contrast to past findings (e.g., Zaiss et al. 2015), these results are in line with recent investigations of Cameroonian, Indonesian, and Liberian volunteers, where a positive correlation was found between infections with soil-transmitted helminths (e.g., *S. stercolaris*, *A. lumbricoides*, *N. americanus*) and arachidonic acid (precursor of pro-inflammatory leukotrienes) pathways, proinflammatory cytokines and T helper lymphocytes (TH1, TH2), confirming an association of helminths with host immune system mechanisms (Rosa et al. 2018; Rubel et al. 2020).

In summary, increased bacterial richness in helminth-positive hosts is sometimes associated with a stable gut environment (i.e., one showing higher resilience and functional redundancy) and potential health benefits, such as reduced inflammation and improved immune regulation. However, other studies report the proliferation of potentially pathogenic microorganisms or the activation of pro-inflammatory pathways in the presence of helminths. These dualistic outcomes may be explained by the ecological complexity of helminth-microbiota-host interactions, driven in part by the differences in host exposure and environmental persistence.

However, in non-human primates, the predominantly observational nature of studies limits our understanding of their role in host health, in contrast to more insightful human studies. Controlled experimental designs and more advanced or specific technologies could help elucidate clearer links between helminth-associated changes in microbial composition and functional pathways and should be integrated into non-human primate research in natural settings.

### Bacteria and fungi

Intestinal fungi represent a much smaller proportion of cells than gut bacteria (Zhang et al. 2022a), yet they play similarly important roles in host health (Hoffmann et al. 2013). For example, in humans, they contribute to mitigating pathogenic infection (Richard and Sokol 2019), interact with immune cells (Domer and Domer 1988; Kozłowska et al. 2020; Levitz et al. 2015), and influence host metabolic activity (Mims et al. 2021). Similarly to bacteria, studies on both human and non-human primates have noted that gut fungal richness and composition are strongly influenced by the environment, diet, and/or ecological adaptations of the host (humans: Auchtung et al. 2018; Black lion tamarins (*Leontopithecus chrysopygus*): Carvalho et al. 2014; Udzungwa red colobus and yellow baboons: Barelli et al. 2020; humans and four distinct species of non-human primates: Sharma et al. 2022; Tibetan Macaques (*M. thibetana*): Sun et al. 2020; rhesus macaques: Taylor et al. 2018; black snub-nosed monkeys (*Rhinopithecus bieti*): Wang et al. 2023; Hainan gibbons (*Nomascus hainanus*): Yang et al. 2022).

Despite their similarly important role in host health, intestinal fungi remain far less studied than gut bacteria, and only a few recent studies have attempted to analyze both gut bacteria and fungi simultaneously to assess their potential interactions. This gap is particularly evident in non-human primates living in natural conditions. One study on captive rhesus macaques suggested that gut fungal composition is influenced more by food sources and environmental exposure than by the host itself (Taylor et al. 2018). However, despite differences in host-symbiont relationships, other studies indicate parallel variations in the abundance of major bacterial and fungal taxa between gut niches. For example, such patterns have been observed in wild cynomolgus monkeys (*M. fascicularis*: Y. Yang et al. 2023) and Udzungwa red colobus monkeys, where direct negative associations were found between specific gut fungal (e.g., *Sordariomycetes* and *Dothideomycetes*) and bacterial classes (e.g., *Clostridia* and *Bacteroidia*) (Barelli et al. 2021). Concomitant variations in bacterial and fungal composition were also observed independently in male and female wild yellow baboons (Bambi et al. 2024).

However, such correlations do not necessarily indicate direct interactions, as parallel variation could result from shared environmental or dietary influences rather than direct microbiota interplay. One study on zoo-hosted western lowland gorillas found that changes in fungal and bacterial composition were likely associated with similar metabolic functions, such as the breakdown of complex carbohydrates. This suggests that different microbial taxa may interact functionally, contributing to overlapping metabolic pathways (Houtkamp et al. 2023).

With regards to the possible impact of associations between these taxa on host health, the only studies in captive non-human primates that report changes in fungi and bacteria are those in association with inflammatory states of the host immune system. For example, a study conducted on cynomolgus macaques (*M. fascicularis*) observed that individuals with ankylosing spondylitis showed a concomitant decrease in gut fungal and bacterial richness (Zhang et al. 2022b). In another study, macaques with chronic diarrhea undergoing fecal microbiota transplantation (FMT), showed a decrease in the abundance of fungi (e.g., *Trichosporon asahii*) and bacteria (e.g., *Cloacibacillus porcorum* and *Desulfovibrio desulfuricans*) associated with an inflammatory state (such as cytokine production) and a concomitant increase in fungi (e.g., *Aspergillus*) and bacteria (e.g., *Lactobacillus fermentum*, *Lactobacillus ruminis* CAG_367 and *Lactococcus raffinolactis*) associated with an anti-inflammatory state (Tian et al. 2022).

These findings align with multiple human studies showing that inflammatory diseases lead to a reduction in both fungal and bacterial richness, along with decreases in specific bacterial taxa considered markers of a healthy gut (e.g., *Lachnospiraceae* and *Bacteroidetes, Ruminococcaceae*). At the same time, these conditions are often associated with increases in potentially pathogenic fungi and bacteria, such as *Candida* or *Clostridium difficile* (Bhute et al. 2017; Chehoud et al. 2015; Henderickx et al. 2024; Hong et al. 2020; Jayasudha et al. 2018; Jiang et al. 2023a; Lemoinne et al. 2020; Liguori et al. 2016; Paterson et al. 2017; Wetzel et al. 2023; Zuo and Ng 2018). More direct evidence of interactions between fungi and bacteria comes from human studies. For instance, the production of peptidoglycan fragments by potentially pathogenic bacteria (such as *Pseudomonas aeruginosa* and *Staphylococcus aureus*) has been shown to promote the transition of *Candida albicans* from the yeast to hyphal form, increasing fungal pathogenicity and contributing to disease progression (Crump 2022).

The relatively few studies on bacteria-fungi associations published thus far suggest that changes in fungal and bacterial richness often occur simultaneously and in the same direction, and that this association may influence metabolism and health. In both non-human and human primates, reduced gut microbial diversity, affecting both fungi and bacteria, has been associated with inflammatory diseases and an increase in pathogenic taxa. These findings reinforce the critical role of fungi in shaping gut microbiome dynamics and highlight their potential as bioindicators of ecosystem health. Furthermore, fungi play a key role in immune regulation, particularly in the context of inflammatory diseases, where shifts in fungal communities may contribute to immune imbalances. Given their sensitivity to environmental changes (Raimondi et al. 2019) and their close links to host immune states, integrating fungal research into conservation research could offer valuable insights into the impacts of biodiversity loss and primate health. Expanding our understanding of these microbial interactions in wild populations may also enhance strategies for wildlife health monitoring and ecosystem management.

### Bacteria and viruses

Gut viruses include bacteriophages, viruses that replicate in the cytoplasm of specific gut bacteria, and eukaryotic viruses that replicate in host cells. The former are the most abundant viruses in the gut and may play a crucial role in maintaining intestinal equilibrium by regulating bacterial mortality (Pargin et al. 2023). That is, when phages transition from a quiescent lysogenic state to an active lytic cycle, the infected bacteria lyse and die (Zuppi et al. 2024).

Given their biology, changes in the composition of bacteriophages are inextricably associated with changes in bacterial composition; however, studies of bacteriophage-bacteria associations and interactions are very few. Interestingly, in captive macaques, following antibiotic treatment, a substantial decrease in gut bacterial richness and relative abundance was followed by the disappearance of one family of bacteriophages present in healthy macaques (Li et al. 2019b), highlighting the association between bacteriophages and bacteria. In addition, concomitant decreases in gut bacteriophage and bacterial richness with age was reported in captive female cynomolgus monkeys (Tan et al. 2021).

This interdependence is equally evident in humans, where studies show how the associations of phages and bacteria exert a mutual influence on host health. For instance, during the transition from infancy to childhood, changes in bacterial communities shape the growth of phages carrying metabolic genes aligned with the nutritional needs of these bacterial populations (Galperina 2024). Human studies have also identified unique bacteriophage-bacteria associations in individuals with specific conditions, such as diabetes subtypes (Poulsen et al. 2024), hepatocellular carcinoma (Jinato et al. 2024), and children with asthma (Leal Rodríguez et al. 2024). Moreover, phages have been shown to indirectly shape the composition of gut bacteria by influencing the production of secondary metabolites, such as SCFAs, secondary bile acids, and branched-chain amino acids, that are crucial for metabolic processes tied to cardiometabolic diseases (Kirk et al. 2024). Collectively, these findings underscore the intricate, reciprocal relationship between bacteriophages and gut bacteria and their profound impact on gut health and disease in both non-human and human primates.

Eukaryotic viruses, on the other hand, have been reported to influence gut bacterial communities by triggering immune responses or altering the gut environment in ways that favor the growth of certain bacterial species over others (Berger and Mainou 2018). However, findings remain inconsistent. For example, alterations in gut bacterial composition and an increase in potentially pathogenic bacteria were been observed in wild gray lemurs (*M. griseorufus*) naturally infected with adenovirus (adenovirus: Schmid et al. 2022; Wasimuddin et al. 2019), wild chimpanzees (*P. troglodytes schweinfurthii*) infected with SIV (Moeller et al. 2013), and captive rhesus macaques experimentally infected with SIV (Glavan et al. 2016; Handley et al. 2016; Johnson et al. 2022; Klase et al. 2015; Marchetti et al. 2013; Russel 2019; Tanes et al. 2021) or SARS-CoV-2 (Chen et al. 2018). Instead, similar changes were not observed in SIV-infected wild chimpanzees or western lowland gorillas (Barbian et al. 2018; Moeller et al. 2015), and in some cases, bacterial richness even increased following viral infection, as reported in captive rhesus macaques (Allers et al. 2020; Hensley-McBain et al. 2016) and wild vervet monkeys (*Chlorocebus pygerythrus*) after experimental SIV infection (Jasinska et al. 2020).

The studies of Wu et al. (2022) highlight contrasting patterns in the relationship between gut microbiota and viral communities in health and disease. They found that in both healthy macaques and those with depressive disorder (a mental disorder marked by persistent sadness, loss of interest, and functional impairment), bacterial and viral diversity changed in synchrony, suggesting an association between these communities, as observed in other mammals (Lima et al. 2019). In contrast, Cao et al. (2024a) reported that in Crohn’s disease, this correlation was disrupted, with bacterial and viral richness varying independently, indicating a dysregulated interaction. These mixed findings suggest that while viral infections can influence gut bacterial composition, the nature and extent of these effects are likely dependent on factors such as host species, viral strain, infection severity, and broader ecological or physiological conditions.

Several factors may explain these contradictory results (Brenchley and Ortiz 2021). One key consideration is the stage of inflammatory response during viral infection, which can contribute to bacterial enrichment or depletion and, consequently, impact associated metabolic functions. For example, earlier stages of infection may cause an increase in bacteria producing SCFAs, which leads to a decrease in the state of inflammation. However, as the infection progresses, SCFA levels may decline, altering immune responses and potentially exacerbating inflammation (Feng et al. 2023). Additionally, long-term co-evolution between certain viruses and their hosts may led to adaptations that help maintain gut bacterial balance, thereby mitigating disease progression (Jasinska et al. 2023; Raehtz et al. 2020). Co-infection and inflammation severity may further contribute to variability in observed effects. For example, in humans, co-infection with HIV and cytomegalovirus has been linked to increased inflammation (Ramendra et al. 2020). Similarly, SARS-CoV-2 infections in humans have had varying effects on gut microbiota (Zuo et al. 2020), potentially influenced by pre-existing conditions or viral activity (Zuo et al. 2021). In non-human primates, differences between captive and wild individuals may also play a role; alterations in gut microbiota following experimental infections in captivity (compared to the lack of observed effects in wild populations) could be influenced to treatment doses or pre-existing microbiota differences, such as the absence of helminths, dietary changes, and reduced exposure to environmental microbiota.

These insights highlight the complexity of virus-microbiota-host relationships and underscore the role of both host and viral factors in shaping these dynamics. This complexity is also evident in the metabolic alterations associated with changes in bacterial richness and composition during viral infection. In cynomolgus and rhesus macaques infected with SARS-CoV-2, increased production of tryptamine, a cytotoxic compound, was noted, potentially aggravating the pathogenic effects of the ongoing infection (Paley 2021; Sokol et al. 2021). Likewise, SARS-CoV-2-infected cynomolgus macaques (Sokol et al. 2021) as well as SIV- and cytomegalovirus-infected rhesus macaques (Chin et al. 2022; Tanes et al. 2021), exhibited reductions in SCFA-producing bacteria and butyrate levels, which may have contributed to increased inflammation and heightened disease severity (but see Santos Rocha et al. 2018).

Interestingly, very recent research on the interaction between SARS-CoV-2 and gut bacterial components in humans found a direct link between the human gut microbiota and viral genome (Cao et al. 2024b). This study discovered that certain mutations in the bacterial mRNA of the human microbiota influences the mutational evolution of SARS-CoV-2 by changing its binding sites, modifying its interactions with host cells and immune responses. These findings, in addition to highlighting the co-evolutionary relationship between bacteria and viruses, provide clues to the implications these interactions may have on host health, adaptation, and disease development.

In conclusion, the intricate relationships between gut bacteriophages, eukaryotic viruses and bacteria are crucial to the health of non-human primates. Our review highlights how bacteriophages, by regulating bacterial populations, contribute to maintaining a balanced gut microbiota composition, which is essential for the overall health and survival of primates. The role of eukaryotic viruses in shaping gut microbiota composition in non-human primates is particularly evident, at least for viruses like SIV and SARS-CoV-2, although results on captive animals do not necessarily reflect responses in wild populations, emphasizing the ecological complexity of these interactions/associations. These results highlight the importance of considering viral-bacterial associations/interactions in primate conservation efforts, especially given the high risk of human-non-human primate transmission from residents or tourists (Jiang et al. 2023b). Such associations/interactions may influence not only the primate immune response but also their resilience to disease. The variability in how different species respond to infections or changes in their gut microbiota underscores the importance of species- and virus-specific studies.

### Possible implications for host health: Inflammatory response and variation of gut microbiota in primates

In addition to changes in gut microbial diversity and composition observed during pathogen infection or disease states, there is growing evidence that these variations are closely linked to the host’s inflammatory response. Inflammation is a protective immune process triggered by adverse stimuli such as injury (e.g., tissue damage) or external agents (e.g., pathogens, irradiation, toxic compounds: (Medzhitov 2010; Sherwood and Toliver-Kinsky 2004). By activating a signaling cascade, producing specific molecules, and recruiting inflammatory cells, the immune system works to isolate and eliminate xenobiotic compounds ultimately promoting tissue healing (Chen et al. 2018). Since the cellular pathways involved in inflammation are shared between the host and gut bacteria and fungi (Cerf-Bensussan and Gaboriau-Routhiau 2010), inflammatory responses can inadvertently alter the richness and composition of intestinal symbionts. These shifts, while part of a protective mechanism, can paradoxically have negative consequences for host health.

Although further research is needed to fully understand the complex communication mechanism between gut microbiota and the host immune system, especially in wild populations of non-human primates, it is increasingly clear that intestinal homeostasis is intrinsically linked to overall health (Cerf-Bensussan and Gaboriau-Routhiau 2010). For example, in the presence of pathogens, certain microbial taxa, such as *Campylobacter* or *Helicobacter* (Westreich et al. 2019) and *Trichomonas*, can proliferate more successfully by suppressing bacterial and fungal taxa with anti-inflammatory properties (Hashimoto-Hill and Alenghat 2021; Li et al. 2019a, b, c; Ost et al. 2017; Ott et al. 2008; Sokol et al. 2017). However, it remains unclear whether the observed decrease in bacterial diversity is directly caused by pathogen invasion or by the inflammation triggered in intestinal tissues. Laboratory studies on humans suggest that several microbial taxa contribute to inflammation, either by up- or down-regulation key immune molecules (Hirao et al. 2014; Montero et al. 2021; Wu and Wang 2019) or through microbial translocation (i.e., the passage of bacteria or bacterial metabolites from the gut into systemic circulation). This phenomenon has been shown to occur during HIV, SIV and SARS-COV-2 infections in both human and non-human primates (Crakes and Jiang 2019; Ericsen et al. 2016; Fakharian et al. 2023; Hirao et al. 2014; Pandrea et al. 2022; Ponte et al. 2016; Roy et al. 2021; Stern et al. 2019; Zevin et al. 2016), indicating that certain bacterial taxa increase in response to infection, potentially modulating immune response. Unfortunately, the effects of antiviral treatments on microbial translocation and inflammation in captive rhesus macaques have been inconsistent. In some cases, antiviral therapy fully resolved inflammation (Siddiqui et al. 2020), while in others, it had no effect, leading to prolonged immune activation (Lavinder et al. 2022). Several studies conducted in both human and non-human primates suggest that specific bacterial genera (e.g., *Campylobacter, Streptococcus, Prevotella, Clostridium, Peptostreptococcus*: Gou et al. 2021; Gu et al. 2020; Lv et al. 2021; McKenna et al. 2008; Montero et al. 2021; Sokol et al. 2021; Tao et al. 2020; Zuo et al. 2020, 2021) and fungal taxa (*Candida*, *Saccaromycetes* and *Aspergillus*: Dheeb 2014) may be associated with increased production of pro-inflammatory cytokines (Dinarello 1997; Ericsen et al. 2016; Poly and Guerry 2008). Although the intricate relationship between the gut microbiota and host immune responses remains the focus of ongoing studies, it seems clear that microbial composition plays a crucial role in both gut health and inflammation. This conclusion underscores the need for further investigation, particularly in wild non-human primates, to fully understand these complex relationships and their implications for overall health.

Other eukaryotic organisms, such as helminths, also play a crucial role in immune regulation and intestinal homeostasis in both humans and non-human primates. For example, rhesus macaques with chronic idiopathic diarrhea showed clinical improvement and reduced signs of IBD after experimental infection with whipworms. These individuals exhibited reduced bacterial attachment to the intestinal mucosa, greater bacterial richness, and a microbial composition more similar to that of healthy controls (Broadhurst et al. 2012). Similarly, human patients with celiac disease experimentally infected with hookworm experienced an increase in gut bacterial richness associated with a more stable composition (Giacomin et al. 2015). Additionally, simultaneous helminth and virus infections have been associated with a decrease in bacteria involved in the production of SCFAs (i.e., butyrate, acetate, propionate and desamino tyrosine) with known anti-inflammatory properties (Chang et al. 2014). SCFAs promote the activity of T-regulatory cells (Arpaia et al. 2013), and enhance host resistance to infections, including lower respiratory tract infections: LRTI: Haak et al. 2018). In a study on SIV-infected captive rhesus macaques, SCFA levels were negatively correlated with viral load, suggesting that higher SCFA concentrations were associated with lower viral replication, possibly due to immune modulation or the maintenance of gut barrier integrity (Johnson et al. 2024).

Overall, these findings highlight the intricate relationships between microbial composition, helminth presence, immune regulation, and infection outcomes. They underscore the anti-inflammatory and antiviral properties of metabolites produced by certain taxa, as well as the inflammatory potential of others. This suggests that targeting microbial composition could be a promising strategy for modulating inflammation and enhancing host resistance to infections.

## Conclusions

This review summarizes the studies conducted in non-human primates where bacteria have been analyzed simultaneously with at least one other gut taxon to understand the extent of associations and interactions between gut components. We conclude that, despite variations in gut diversity, composition, and metabolic pathways in response to parameters such as disease, habitat or diet, gut organisms may also vary in abundance or composition in association with a second taxon, suggesting they often interact directly with each other. However, very few consistent patterns of directional change have been confirmed across species or taxa. One exception is that the presence of *Entamoeba* spp. which has been associated with increased richness and relative abundance of beneficial bacteria. The distinct patterns of microbial changes observed across different hosts (both individuals and species) likely stem from intrinsic factors, microbial community dynamics, or their co-evolutionary history. Moreover, associations and interactions between gut taxa appear be influenced by host pathological conditions and environmental factors, such as captivity, diet, and geographical location.

In disease states or during inflammatory responses, where only a limited number of studies have explored gut taxa relationships, changes in gut flora seem to play a role in maintaining gut homeostasis. For example, SFCA-producing bacteria help regulate eukaryotic viruses and other pathogen proliferation, while bacteriophages modulate bacterial populations. Additionally, both helminths and fungi appear to interact with bacteria to control the inflammatory response.

However, most studies on non-human primates have been observational rather than experimental, relying on targeted metagenomic techniques (amplicon sequencing) on fecal samples. While this approach provides rapid assessments of taxonomic diversity, it typically resolves taxa only to genus level, allowing for only basic predictive analyses of associated metabolic pathways. We suggest that species or strain-level analyses using untargeted metagenomics would provide a more detailed assessment of potential function of the gut microbiome (i.e. based on genetic diversity) by revealing the specific pathways involved in gut taxa interactions, especially during host inflammatory responses.

Importantly, moving beyond purely descriptive approaches will require the adoption of field-friendly functional tools. For instance, fecal calprotectin and neopterin assays, cytokine profiling would allow assessment of inflammation in wild primates under natural conditions. In addition, integrating parasitological measures (e.g., parasite load estimated via fecal flotation or molecular metabarcoding techniques) with microbial and immunological data would enable a more comprehensive understanding of host health. Coupling these approaches with behavioral observations (such as grooming networks or ranging patterns) and ecological parameters (habitat quality, food availability, anthropogenic disturbance) would provide a clearer roadmap for disentangling the complex links between host immunity, microbial interactions, and environmental context.

Finally, most direct interactions between bacteria and other gut taxa discussed in this review have been primarily reported in humans. This highlights the complexity of this ecosystem and suggests that further research on non-human primates could improve our understanding of host health. From a conservation perspective, a greater awareness of gut microbial dynamics could serve as a valuable indicator of the well-being of non-human primates, many of which are threatened or endangered. Anthropogenic impacts on their health could also be assessed by examining the richness and nature of microbial interactions. A more detailed understanding of these dynamics and their association with the host immune system could help identify relevant biomarkers for improved monitoring and conservation efforts. Finally, a deeper comprehension of these interactions would enhance our understanding of host ecology and have important implications for evolutionary studies on the relationships between hosts and their complex microbial communities.

## Supplementary Information

Below is the link to the electronic supplementary material.

Supplementary file1 (XLSX 24 kb)

Supplementary file2 (XLSX 20 kb)

Supplementary file3 (XLSX 61 kb)
